# Dietary fiber inulin mitigates peripheral neuropathy in different stages of diabetes via modulating gut microbiota and metabolites in *db/db* mice

**DOI:** 10.1371/journal.pone.0336962

**Published:** 2025-11-20

**Authors:** Ke Li, Wenjun Fu, Yuanyuan Liu, Jing Xue, Xiaoli Yang, Leiwen Wang, Jin Xu, Junbai Ma, Rong Su, Xia Yang, Yuning Sun, Hao Wang

**Affiliations:** 1 School of Basic Medical Sciences, Ningxia Medical University, Yinchuan, Ningxia, China; 2 People’s Hospital of Ningxia Hui Autonomous Region, Yinchuan, Ningxia, China; 3 Sichuan Vocational College of Health and Rehabilitation, Zigong, Sichuan, China; 4 The First Clinical Medical College, General Hospital of Ningxia Medical University, Yinchuan, Ningxia, China; 5 Department of Biochemistry and Molecular Biology, School of Basic Medical Science, Ningxia Medical University, Yinchuan, Ningxia, China; 6 Department of Pathogenic Biology and Medical Immunology, School of Basic Medical Sciences, Ningxia Medical University, Yinchuan, Ningxia, China; Universite du Quebec a Montreal, CANADA

## Abstract

The pathogenesis underlying diabetic peripheral neuropathy (DPN) remains largely elusive. Due to current unsatisfactory therapeutic approaches, new strategies for the control of DPN are needed. The present study was designed to assess whether inulin could serve as a potential neuroprotection against DPN in diverse stages of diabetes. Leptin receptor-mutant *db/db* mice were used as a model for DPN to dynamically assess the effects of inulin on DPN in diverse diabetic groups. According to blood glucose, the mice were randomly divided into prediabetes group (PDM group), inulin treated prediabetes group (INU/PDM group), diabetes group (DM group) and inulin treated diabetes group (INU/DM group). After 6 weeks of treatment, we found that inulin supplementation attenuated the neuropathic phenotypes in PDM and DM, including mechanical allodynia, thermal hyperalgesia and nerve conduction. Furthermore, inulin administration remarkably suppressed the levels of pro-inflammatory IL-6, TNF-α, and IL-17A in diverse diabetic groups with DPN, but increased an anti-inflammatory IL-10 in INU/PDM group, suggesting that dietary inulin intervention may suppress the DPN inflammation in different diabetic stages. Moreover, inulin supplementation markedly reduced the circulating LPS translocation. Metabolomics analysis revealed that inulin treatment significantly modulated the levels of 8 stage-specific metabolites; notably, it increased anti-inflammatory, anti-diabetic and neuroprotective metabolites taurine and dodecanoic acid in prediabetic mice, while decreasing pro-inflammatory mediators including oleamide and adrenic acid. In diabetic mice, inulin elevated metabolites including methylation (S-Adenosylmethionine), glucose homeostasis (glucose 6-phosphate), N-acetyl-L-phenylalanine and quinate. These metabolites are implicated in pathways such as bile acid metabolism, fatty acid oxidation, and neurotransmitter regulation, suggesting that inulin may exert neuroprotective effects by restoring metabolic homeostasis in a stage-dependent manner. Furthermore, rectification of gut dysbiosis by dietary inulin administration, with a major impact on increasing intestinal beneficial bacteria |(*Bacteroides* and *Cyanobacteria*) and decreasing pro-inflammatory bacteria (*Ruminiclostridium_6*, *Mucispirillum, Deferribacteres* and *Tenericutes*), exerted a close and complex interactions with metabolites, inflammatory factors, and peripheral neuropathic indicators. Collectively, dietary inulin intervention ameliorated DPN via reshaping gut microbial metabolism and suppressing inflammation, which may potentially provide an effective and safe therapeutics for the control of the disease.

## 1. Introduction

Unhealthy diet, sedentary lifestyle and an aging population have rapidly led to an increase in the occurrence and progression of type 2 diabetes mellitus (T2DM), placing a substantial burden on individuals, society and the economy [1]. Diabetic peripheral neuropathy (DPN) is the most common long-term complication of diabetes, ultimately leading to an elevated incidence of lower-limb amputation and even death. Hyperglycemia-induced diffuse damage to peripheral nerve fibers results in demyelination, axonal degeneration and peripheral neuritis, which are clinically manifested by allodynia and hyperalgesia [2]. Growing evidence suggests that peripheral neuropathy is common in prediabetes (25%) and diabetes (at least 50%) [3,4]. Due to limited methods for the management of DPN apart from optimizing glycemic control, more strategies for the prevention and treatment are needed.

Accumulating studies have indicated that gut dysbiosis plays a vital role in the development of numerous chronic metabolic and neurological disorders, including obesity, T2DM, neuroinflammation and neurodegenerative diseases [5]. Abnormal gut metabolites derived from gut dysbiosis and disruption of the gut barrier are closely associated with hyperglycemia metabolism and insulin sensitivity [6,7]. Emerging study has suggested that gut microbiota dysbiosis is implicated as a critical factor for DPN pathogenesis [8]. Moreover, a randomized clinical trial showed that transplanting gut microbiota from healthy donors to patient with DPN can rectify gut microbiota disorders, increase beneficial short-chain fatty acids (SCFAs), reduce metabolic inflammation, as well as effectively alleviate peripheral neuropathy [9]. After unveiling the underlying role of gut microbiota in T2DM and its neuropathy, we hypothesized that reshaping homeostasis of gut microbiota and metabolites might be a potential new strategy for preventing and treating diabetic neuropathy.

Chronic low-grade inflammation is prominently involved in the pathogenesis of diabetes-related complications [10]. Dysbiosis of gut microbiota results in a pro-inflammatory micro-environment and systemic endotoxemia, both of which are implicated in the pathogenesis of neuropathy and metabolic disorders [11]. Lipopolysaccharide (LPS), an important virulence factor from Gram-negative bacteria, triggers an inflammatory response in the host organism by binding to Toll-like receptor 4 (TLR4). This interaction induces the activation and sensitization of nociceptive neurons [12]. Studies in rodent models of diabetic peripheral nerve injury have indicated that blocking pro-inflammatory interleukin-6 (IL-6) and tumor necrosis factor-α (TNF-α) expressions attenuates neuroinflammatory response and pathological damage [13,14]. Furthermore, a recent study in both rodent and humans validates that the modulation of gut microbial may contribute to the amelioration of DPN [9]. Therefore, the chronic low-grade inflammation regulated by the gut microbiota may be a major therapeutic potential for prevention and treatment of DPN.

Dietary fiber in variety of foods is considered as an essential nutrient for humans [15]. Inulin is an important kind of dietary fibers that stands for a naturally occurring fructan derived from plant-based vegetables such as chicory, burdock root, onion, garlic, and leek. Through industrial processing, it is commercially produced as inulin-type fructans [16,17]. It is widely utilized in the food industry as a functional ingredient to replace sugar and fat, enhancing nutritional profiles whereas reducing caloric content. Dietary supplementation with inulin has been demonstrated to show significant potential benefits for the management of metabolic disorders, including insulin resistance [18], T2DM [19], and cardiometabolic diseases [20]. Its efficacy is mainly attributed to mechanisms such as the modulation of gut microbiota composition, plasma metabolome(e.g., SCFAs, bile acids and amino), and reduction of systemic inflammation [21]. More importantly, these metabolites can also enter the circulation and regulate the release of anti-inflammatory factors (e.g., interleukin-10 (IL-10)) by immune cells, depending on the inflammatory micro-environment [22]. Inulin promotes the homeostatic remodeling of colonic epithelium through the γδT cell-IL-22 axis via regulating gut microbiota and its metabolites [23]. Our previous study reported that inulin supplementation ameliorated diverse stages of T2DM via suppressing inflammation and modulating gut microbiota, especially in prediabetic and early diabetic stages [24]. However, the evidence for a protective role of dietary inulin in diabetic peripheral neuropathy remains poorly explored. We therefore hypothesize that dietary inulin supplementation may ameliorate diabetic peripheral neuropathy through the modulation of gut microbiota and their metabolite profiles, leading to reduced systemic inflammation and enhanced neuroprotection across different stages of diabetes.

## 2. Materials and Methods

### 2.1. Murine model and experimental design

Leptin receptor-mutation *db/db* mice has been extensively studied as a well-characterized murine model of DPN [25,26]. In this study, we used only female db/db mice based on previous evidence indicating that female diabetic mice show more consistent peripheral neuropathy phenotypes and lower behavioral variability than male mice, which enhances reproducibility and signal resolution for microbiota-metabolite-host axis studies [27]. Four-week-old female db/db mice (Vital River Laboratory Animal Technology Co., Ltd, Beijing, China) were adopted to generate a DPN model. After 1 week of adaptive feeding, the fasting blood glucose (FBG) levels were examined every 4 days for a total of 3 times. Mice were fasted for 10 h and an oral glucose tolerance test (OGTT) was performed. Based on previous studies [24,28], mice with FBG < 7.0mmol/L, glucose load (2-h plasma glucose): ≥ 7.8 and < 11.1 mmol/L were defined as prediabetes group (PDM group, n = 20). In addition, mice with FBG > 7.0mmol/L or glucose load > 11.1 mmol/L more than 3 tests times were defined as diabetes group (DM group, n = 20) [24]. OGTT was repeated independently 3 times. Ten mice in the PDM group or the DM group were accordingly divided into inulin treatment of PDM group (INU/PDM) or inulin treatment of DM group (INU/DM). All groups of mice were fed a standardized diet (kcal %: 10% fat, 20% protein, and 70% carbohydrate; 3.85 kcal/gm) that was obtained from Laboratory Animal Center of Ningxia Medical University. Inulin in the intervention derived from commercial soluble dietary fiber powder (ViolfTM, BAHEAL Medical Inc., Qingdao, China) containing 91% inulin-type fructan and 9% mixture of sucrose, fructose, and glucose, was orally administered 5% (w/v) to the treated groups (n = 10), as well as control groups were administered with an equal volume of sterile saline (n = 10) [29]. Inulin or vehicle (saline) was administered every day by gavage for 6 weeks. At the endpoint of intervention, fresh fecal samples were obtained and immediately frozen at −80 °C for subsequent microbiome analysis. Thereafter, mice were humanely euthanized by deep anesthesia through the isoflurane inhalation and associated indicators were subsequently investigated. All euthanasia procedures were consistent with the recommendations of the American Veterinary Medical Association (AVMA), and the animal protocols were approved by the Ethics Committee of People’s Hospital of Ningxia Hui Autonomous Region, Ningxia Medical University (No. 2020-NZR-012). Blood samples were collected in EDTA tubes and centrifuged (400 × *g* for 15 min) to obtain plasma samples that were stored at −80 °C until use for the following measurements.

### 2.2. Evaluation of basic metabolic indicators, mechanical allodynia and thermal hyperalgesia

Metabolic monitoring included weekly measurements of ad libitum water intake (graduated bottle method) and cumulative food consumption (pre/post-weight of standard chow). Body weight was monitored weekly using a calibrated electronic balance. Concurrently, FBG levels were assessed weekly via tail vein sampling with a glucometer (One Touch Profile, Johnson & Johnson, Inc. Milpitas, CA, USA). At the end of treatment, plasma insulin concentrations were quantified utilizing a mouse insulin ELISA kit (Wuhan Elabscience Biotechnology Co.,Ltd, Wuhan, China). Concurrently, glycated hemoglobin (GHb) levels were measured using the Mouse Glycosylated Hemoglobin ELISA Kit (Wuhan Genesmei Technology Co., Ltd, Wuhan, China). Plasma total cholesterol (TC) and triglycerides (TG) were quantiatively determined using AU400 automatic biochemical analyzer (Olympus, Tokyo, Japan), as previously described in our established methodology [24].

Behavioral testing for mechanical allodynia was assessed by using von Frey filament stimuli to ipsilateral hind every week as previously described [30]. Mice were adapted in organic glass cages on a metal grid for 30 min prior to the test. Von Frey filaments (0.02-2.0g bending force, North Coast Medical, Inc., San Jose, California, USA) were vertically applied to the plantar surface of the right hind paw. Abrupt paw withdrawal or licking was regarded as positive responses. The 50% paw withdrawal threshold (PWT) was estimated by “up and down” approach [31].

Thermal hyperalgesia was quantified by a radiant heat stimuli plantar device (Chengdu Technology & Market Co., Ltd., Sichuan, China) to detect the paw withdraw latency (PWL) [32]. The measurement was taken at room temperature (20–25 °C) and quiet environment. Each mouse was exposed in a plexiglass cylindrical enclosure for 20 min to habituate to their surroundings. A beam radiant heat source was focused onto ipsilateral plantar surface of the right hind paw. Paw withdrawal latency defined as the duration of time that animal lifted or licked the paw was recorded.

### 2.3. Neurophysiological examination

After evaluating the aforementioned behavioral parameters, the BL-420F biological function experimental system (Chengdu Technology & Market Co., Ltd., Sichuan, China) was used to record and analyze the neurophysiological indices after 6 weeks of inulin intervention.

Mice were anesthetized by intraperitoneal injection of 1% pentobarbital sodium and body temperature maintained at 37 °C. Sciatic-tibial sensory nerve conduction velocities (SNCV) were assessed via stimulating a distal (ankle) and a proximal (sciatic notch) site along the nerve with bipolar needle electrodes (BL-420f biological function determination system; frequency 0.10 Hz, duration 0.2ms, supramaximal stimulus 1V). Nerve conduction velocities were calculated in meters per second from distal and proximal latencies. The sensory nerve action potential (SNAP) amplitudes were tested from peak to peak.

### 2.4. Electron microscopy evaluation

Mice were anesthetized by intraperitoneal injection of 1% pentobarbital sodium and transcardially perfused (4% paraformaldehyde). Sciatic nerves were harvested and fixed with 4% paraformaldehyde at 4 °C for 24h. Sciatic nerve segments were soaked in 2% osmium tetroxide at 4 °C for 2h and rinsed twice with 0.1M phosphate buffer at 15 min intervals. After dehydration in graded series of propylene oxide, the samples were embedded in epoxy resin. Ultrathin sections were cut at approximately 60–70 nm thickness using an ultramicrotome and collected on copper grids. The sections were then double-stained with uranyl acetate for 20 minutes followed by lead citrate for 5 minutes.The morphological changes of the sciatic nerve were evaluated under an H-7650 electron microscope (×2000) (Hitachi, Tokyo, Japan).

### 2.5. Immunohistochemistry

For immunohistochemical analysis, paraffin sections were dewaxed and boiled in 10mM citrate buffer pH6.0. Sections were washed in ddH_2_O and then immersed in 0.3% H_2_O_2_ for 10 min. After washing with PBS, the sections were blocked with 5% normal goat serum for 1h, and incubated with primary antibody mouse (myelin basic protein, MBP 1:200, ab62631, Abcam; myelin protein zero, P0 1:200, ab31851, Abcam) overnight at 4 °C. Tissue sections were washed 3 times with PBS and incubated with second antibody goat anti-mouse (1:100, ThermoFisher) and goat anti-rabbit (1:100, ThermoFisher) for 1h at 37 °C, respectively. The results were observed by an inverted microscope using DAB as a chromogenic reagent.

### 2.6. Intraepidermal nerve fiber density (IENFD)

Hind footpad skins were fixed in 4% paraformaldehyde at 4 °C for 24h, and then immersed in 30% sucrose until they sank. Then, the samples were sliced at 14µm on a freezing sledge microtome. Sections were incubated with primary antibody protein gene product 9.5 (PGP 9.5, 1:200, Sigma-Aldrich, AB1761-I,Singapore) overnight at 4 °C, and with secondary anti-body Alexa Fluor 488 goat-rabbit at room temperature for 1h. Fluorescent images were captured with a Leica DM6 fluorescence microscope.

### 2.7. Determination of circulatory inflammation

Plasma LPS levels in each group were assessed by the limulus amebocyte lysate kit (Xiamen Bioendo Technology Co. Ltd., Xiamen, China) and determined to be < 1.0EU/mL. Circulatory inflammatory cytokines were determined by using BD^TM^ cytometric bead array (CBA) Mouse Inflammatory Cytokine Kit (BD Bioscience, CA, USA). The limit of detection is 0.03 to 5000pg/mL. The detection methods were performed as previously described by our lab [24].

### 2.8. 16S rRNA gene processing and analysis

For characterization of bacterial species, we collected fecal samples for 16S rRNA sequencing as described in a previous study [33]. In brief, the genomic DNA of fresh fecal samples was extracted using cetyltrimethylammonium bromide (CTAB) method. (CTAB) method. The V3 and V4 16S rRNA hypervariable regions were amplified by PCR using primers (341F/806R). Then, the mixture PCR products were purified with GeneJET Gel Extraction Kit (Thermo Scientific). Sequencing libraries were generated according to TruSeq® DNA PCR-Free Sample Preparation Kit for library construction. The library quality was assessed on the Qubit＠ 2.0 Fluorometer (Thermo Scientific) and Agilent Bioanalyzer 2100 system. After library construction, samples were quantified using Qubit and subjected to quality control. Qualified libraries were then sequenced on the Thermo Fisher Scientific Ion S5™ XL platform to generate single-end reads. The raw sequencing reads were processed using Cutadapt to trim low-quality bases and adapter sequences. Reads were demultiplexed according to their unique barcodes, and both barcode and primer sequences were removed to yield initial raw reads. Chimeric sequences were identified and removed by comparing the reads against the reference database using the UCHIME algorithm, resulting in high-quality clean reads for downstream analysis. All clean reads from all samples were clustered into operational taxonomic units (OTUs) at 97% identity threshold using Uparse software. SILVA SSU rRNA databas was used for Taxonomic annotation. Finally, microbial community complexity and structure analysis was conducted using QIIME and R software.

### 2.9. Metabolome analysis

#### 2.9.1. Sample preparation.

The liquid chromatography mass spectrometry (LC/MS) was conducted for untargeted metabolomics analysis. Plasma samples after thawing were extracted by 400 μL methanol, vortexed and centrifuged at 12,000 × g for 10 min at 4°C. Then, 150 μL of 2-chloro-l-phenylalanine (4 ppm) solution was added with prepared 80% methanol water to redissolve the sample, filter the supernatant by 0.22μm membrane and transfer into the detection bottle for LC-MS detection. Metabolites that distinguished between diverse groups were identified based on variable importance in the projection (VIP) values exceeding 1, P-values below 0.05, and q-values for the false discovery rate (FDR) less than 1.

#### 2.9.2. Mass spectrum conditions.

Mass spectrometric detection of metabolites was performed on Orbitrap Exploris 120 (ThermoFisher Scientific, USA) with ESI ion source. Simultaneous MS1 and MS/MS (Full MS-ddMS2 mode, data-dependent MS/MS) acquisition was used. The parameters were as follows: sheath gas pressure, 40arb; aux gas flow, 10arb; spray voltage, 3.50kV and −2.50kV for ESI (+) and ESI (**-**), respectively; capillary temperature, 325 °C; MS1 range, m/z 100–1000; MS1 resolving power, 60000 FWHM; number of data dependent scans per cycle, 4; MS/MS resolving power, 15000FWHM; normalized collision energy, 30%; dynamic exclusion time, automatic.

## 3. Statistical analysis

All experimental data were statistically analyzed with Prism 6.01 (GraphPad Software Inc., CA, USA). The results were represented as mean ± SEM. In general, following the assessment of normality for data, two groups were compared using the unpaired t or Mann-Whitney U test. Differences were analyzed using one-way or two-way ANOVA for multiple groups comparisons. All correlation analyses was performed using Hierarchical All-against-All association testing. *P* < 0.05 was deemed statistically significant.

## 4. Results

### 4.1. Inulin supplementation attenuated the basic metabolic indicators and neuropathic phenotypes in diverse stages of diabetic mice

**Fig 1:**
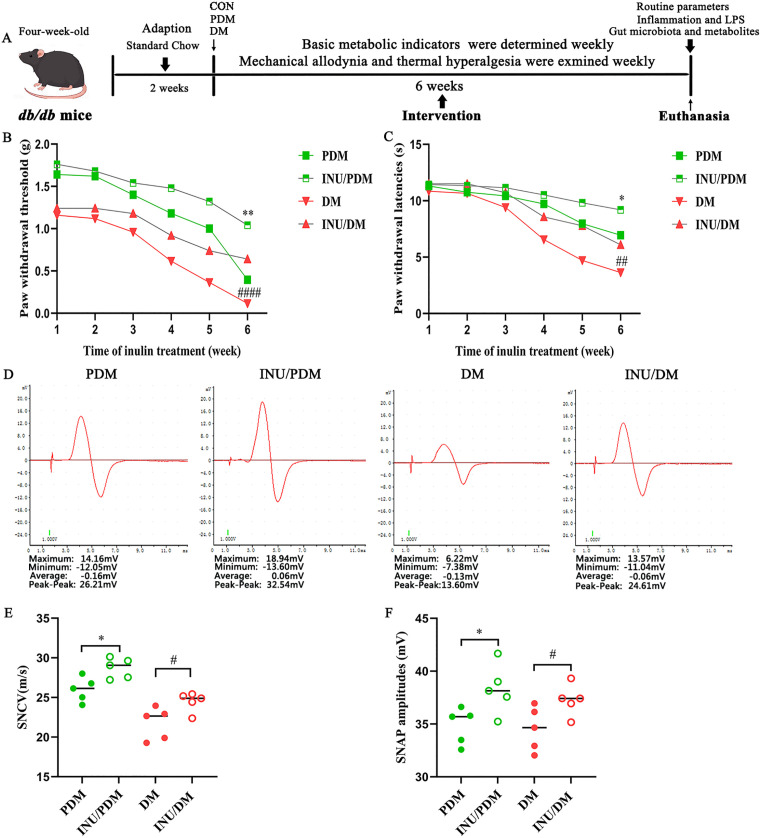
The effects of inulin on allodynia, thermal hyperalgesia and electrophysiological characterization of sciatic nerve in animals with DPN. A: Schematic timeline of the experimental design. B: Effect of inulin treatment on mechanical allodynia in von Frey test. C: Assessment of inulin treatment on thermal hyperalgesia in diabetic neuropathic pain. The paw withdrawal mechanical threshold (B) and paw withdrawal thermal latency (C) were measured at different time points (week 1, 2, 3, 4, 5, 6). D: Representative traces showing the dynamic changes in compound action potential (CAP) of the sciatic nerve in db/db mice during the 6-week intervention period. E: Inulin improved sensory nerve conduct velocity (SNCV). F: Inulin exerted a promotion on sensory nerve action potential (SNAP). Sensory nerve action potential (SNAP) amplitudes recorded at 6 weeks post-intervention. Data are expressed as mean ± SEM, n = 5-10 per group. A two-way ANOVA was used to evaluate significant differences in Figure (B) and (C), while an unpaired Student’s t-test was performed to compared the inulin-treated and untreated groups during the same period in Figure (E) and (F). *P < 0.05 and **P < 0.01 compared with the PDM group; ##*P* < 0.01 and ####*P* < 0.0001 compared with the DM group.

Our investigation demonstrated that dietary supplementation with inulin effectively attenuated body weight gain, hyperglycemcia, dyslipidmia and insulin levels in murine models of prediabetes and diabetes. But the intervention exhibited no statistically different impact on ad libitum water intake and cumulative food consumption during the same period (S1 Table). The effect of inulin on mechanical allodynia and thermal hyperalgesia in diabetic neuropathic pain was depicted as Fig 1. Firstly, the *db/db* mice exhibited characteristic neuropathic phenotypes distinct from C56BL/6J controls, including reduced nerve conduction velocity and elevated thermal hypersensitivity, confirming successful modeling of DPN in both prediabetes and diabetes groups (S1 Fig). Notably, compared to untreated respective model groups (PDM group and DM group), inulin supplement significantly ameliorated this sensory impairment by restoring mechanical sensitivity in INU/PDM group and INU/DM group (Fig 1A). We found that initial PWL value showed no notable discrepancy among these groups, but during the process of treatment, inulin dramatically increased the latency to a heat stimulus in the INU/PDM and INU/DM groups compared with untreated groups (Fig 1B). Moreover, compared with the prediabetes and diabetes groups, the inulin intervention improved the electrophysiological parameters, especially in inulin-treated prediabetes group (Fig 1C). Nerve conduction measurements showed that sensory nerve conduction velocity (SNCV, Fig 1D) and sensory nerve action potential (SNAP, Fig 1E) in the sciatic nerve of untreated groups were visibly reduced after inulin intervention. Taken together, these results indicated that inulin administration could ameliorate the damage of myelinated nerve fibers.

### 4.2. Inulin attenuated the pathological damage of sciatic nerve

**Fig 2:**
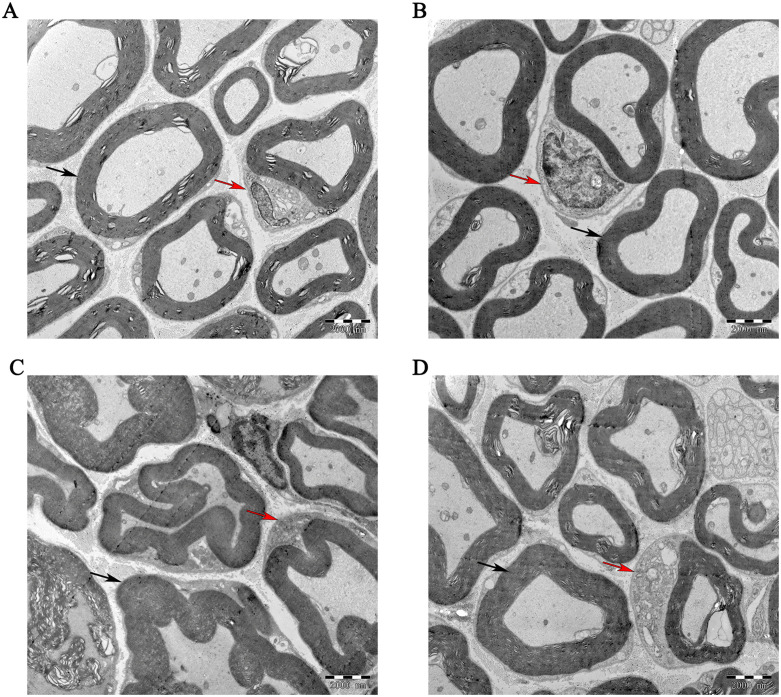
Inulin treatment attenuated the progression of sciatic nerve pathological damage. Histological examination using transmission electron microscopy analysis of the sciatic nerve. Representative TEM images show ultrastructural pathology of the sciatic nerve in prediabetic and diabetic mice, as well as the morphological changes following inulin treatment. A: PDM group. B: INU/PDM group. C: DM group. D: INU/DM group. The red arrow refers to Schwann cell, while the black arrow stands for myelin lamina. Bar × 2000μm. n = 5 per group.

Next, to investigate the impact of inulin administration on the pathological damage to sciatic nerve in mice with DPN, electron microscopy evaluation was utilized for direct observation. In comparison with the PDM group (Fig 2A), the sciatic nerve myelin in the INU/PDM group (Fig. 2B) exhibited a distinct concentric circular laminar structure characterized by a bright and dark pattern with a regular arrangement. Notably, less axonal lesions was found during the PDM stage. In the non-intervention DM group (Fig 2C), the myelin sheath of sciatic nerve was swelled and deformed, accompanied by localized vacuolar degeneration and a loss of the characteristically concentric light and dark structure. The lamellar structure of the myelinated fibers appeared delaminated and distorted in diabetic mice, with some axons displaying separation and edema. Conversely, inulin intervention attenuated the sciatic nerves damage by reducing edema and vacuolar deformation of the myelinated fibers (Fig 2D).

### 4.3. Inulin facilitated the expression of myelin proteins in sciatic nerve

**Fig 3:**
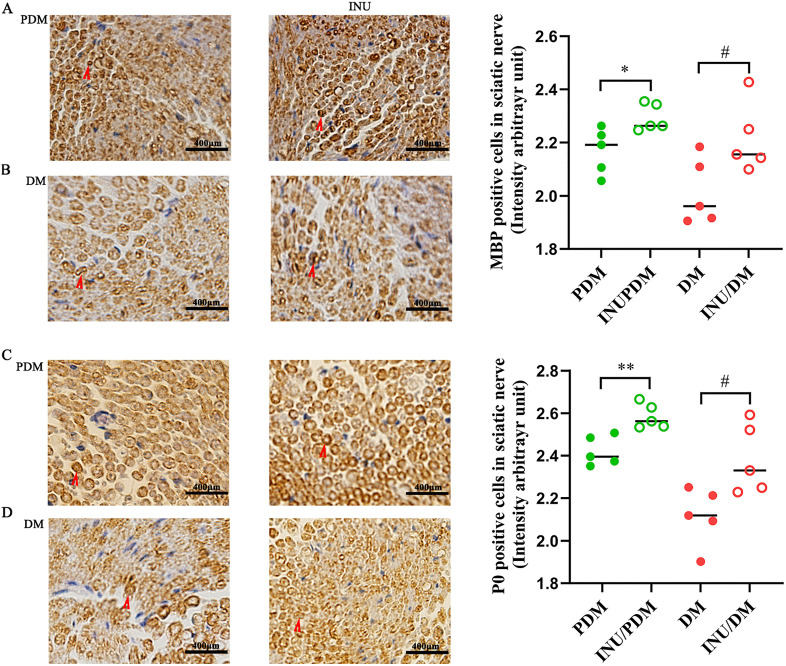
Immunostaining analysis of MBP and P0 at sciatic nerve *in situ.* Left side of A: MBP expression in PDM group; right side of A: MBP expression in INU/PDM group. Left side of B: MBP expression in DM group; right side of B: MBP expression in INU/DM group. Left side of C: P0 expression in PDM group; right side of C: P0 expression in INU/PDM group. Left side of D: P0 expression in DM group; right side of D: P0 expression in INU/DM group. Original magnification × 400. Data are expressed as mean ± SEM; n = 5 per group; unpaired Student’s t-test. *P < 0.05 and **P < 0.01 compared with the PDM group; ^#^*P* < 0.05 compared with the DM group.

MBP and P0, two essential protein components of the myelin sheath, were investigated to assess the integrity of myelin sheaths. Compared to the corresponding model groups, the expression of MBP in the inulin intervention groups including INU/PDM and INU/DM (refer to the right side of Fig 3A and B) was notably elevated in the sciatic nerve. Concomitant with the aforementioned observations, immunostaining of the sciatic nerve *in situ* revealed a notable reduction of P0 protein level in PDM group (depicted on the left side of Fig 3C), which was significantly ameliorated following inulin treatment (illustrated on the right side of Fig 3C). In parallel, this myelin protein P0 also displayed similar expression patterns in diabetic stage (Fig 3D).

### 4.4. Inulin attenuated intraepithelial nerve fibers loss by increasing IENFD expression

**Fig 4:**
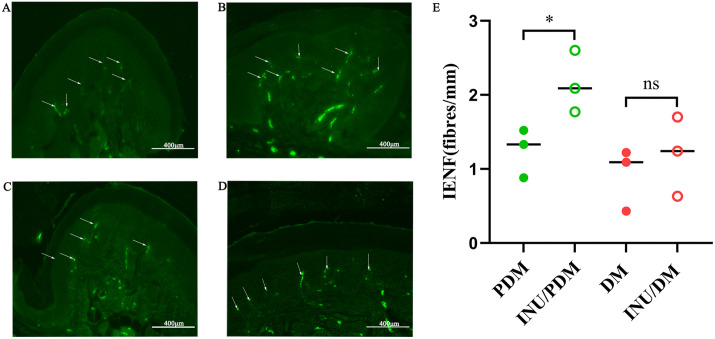
The measurement of IENFD in the plantar skin of mice in diverse groups. Intraepidermal nerve fiber density (IENFD) assessment and representative immunohistochemical images of plantar skin. Representative images of PGP9.5-stained intraepidermal nerve fibers (IENFs, indicated by white arrows) in the plantar skin from the **(A)** PDM group, **(B)** INU/PDM group, **(C)** DM group and **(D)** INU/DM group. **(E)** Quantification of IENFD across groups. Original magnification × 400. Data are presented as mean ± SD. n = 3 per group. Unpaired Student s t-test was used for statistical analysis.

We conducted an evaluation peripheral neuropathy by assessing IENFD in the footpads of mice in diverse groups (Fig 4). Compared to untreated PDM group, prediabetic mice receiving inulin intervention (INU/PDM group) exhibited increased IENFD (Fig 4A and 4B). However, this phenomenon is not prominent in the diabetic stage (Fig 4C and 4D). These results (Fig 4E) indicated that inulin supplementation effectively mitigated small nerve fiber damage associated with prediabetic stage of diabetes.

### 4.5. Inulin may protect against diabetic peripheral neuropathy by restraining inflammation

Inflammation has been identified as a significant factor in the onset and progression of DPN. To further elucidate the potential anti-inflammatory effects of inulin in the neuroprotection, we examined a series of inflammatory indicators in the peripheral blood of each group. Peripheral circulating inflammatory indicators were given in Table 1. As a result, the level of LPS in plasma was markedly decreased in the diverse stages of diabetic mice after inulin treatment. Pro-inflammatory IL-6 and TNF-α were dramatically lower in different stages following inulin administration (INU/PDM group and INU/DM group) than those in respective untreated model groups. The concentration of peripheral interleukin-17A (IL-17A) was distinctly elevated in DM group compared with that in inulin intervention (INU/DM group), although no significant difference was observed between prediabetes groups (PDM group and INU/PDM group). Additionally, anti-inflammatory IL-10 showed a significant increase in the INU/PDM group, but exhibited no change in INU/DM group, suggesting that IL-10 may play a critical role in the negative regulation of inflammation during the prediabetic stage.

**Table 1:** Effects of inulin on plasma inflammatory indicators.

	LPS (EU/mL)	IL-6 (pg/mL)	TNF-α (pg/mL)	IL-17A (pg/mL)	IL-10 (pg/mL)
PDM group	0.193 ± 0.012	17.480 ± 4.555	22.260 ± 7.502	2.176 ± 0.929	21.960 ± 3.539
INU/PDM group	0.166 ± 0.010**	9.942 ± 4.536*	11.520 ± 4.891*	1.532 ± 0.553 ^ns^	29.380 ± 4.179*
DM group	0.414 ± 0.012	28.590 ± 6.369	30.930 ± 3.083	4.962 ± 0.886	28.960 ± 4.371
INU/DM group	0.381 ± 0.022^#^	17.480 ± 6.662^##^	19.280 ± 4.291^##^	3.352 ± 0.735^#^	35.070 ± 6.352 ^ns^

Data are presented as means ± standard deviation; *P < 0.05 and **P < 0.01 compared with the PDM group; #*P* < 0.05 and ##*P* < 0.01 compared with the DM group; ns: no significance; n = 5 per group; unpaired Student’s t-test.

**Fig 5:**
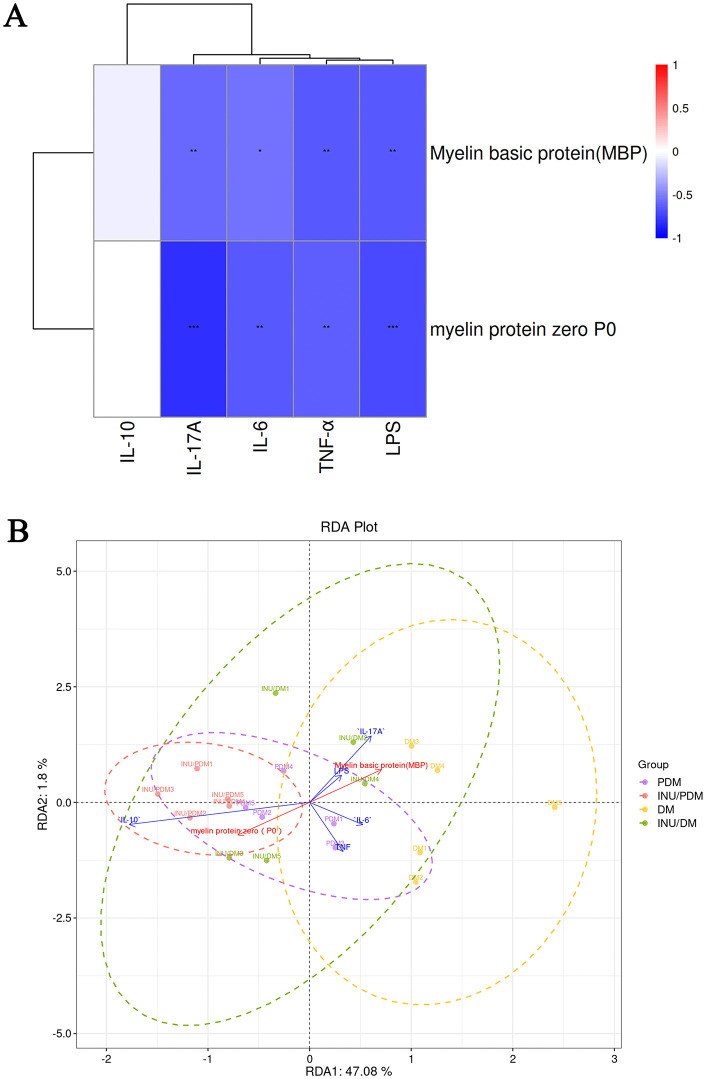
Diabetic peripheral neuropathy is associated with pro-inflammatory/anti-inflammatory cytokines and myelin proteins. A: Correlation matrix focusing on the pro-inflammatory/anti-inflammatory cytokines and myelin proteins. B: Redundancy analysis (RDA) indicating the interaction between myelin proteins and pro-inflammatory/anti-inflammatory cytokines in the context of peripheral neuropathy. *P < 0.05, **P < 0.01, ***P < 0.001. n = 5 per group.

Furthermore, we demonstrated the correlation between inflammatory indicators and myelin proteins (Fig 5). MBP was negatively correlated with pro-inflammatory IL-6, IL-17A, TNF-α, and LPS (Fig 5A). There was no association between IL-10 and myelin proteins. A similar comparable trend was observed in the correlation analysis between P0 and aforementioned four factors, indicating the pro-inflammatory factors may contribute to the development of peripheral neuropathy. Meanwhile, we also used redundancy analysis (RDA) to verify inflammatory indicators association with myelin protein (Fig 5B). RDA was performed using the vegan package (v4.0.3) in R, a multivariate statistical employed to visualize the relationships between dependent variables (myelin proteins) and a set of explanatory variables such as aforementioned pro-inflammatory/anti-inflammatory cytokines. RDA revealed that 47.8% of the variation was explained by axis 1 (RDA1) while 1.8% of the variation was described by axis 2 (RDA2). These variables showed that nearly 50% of the variation in the data, including pro-inflammatory IL-6, IL-17A, TNF-α, and LPS, contributed significantly to peripheral neuropathy.

### 4.6. Inulin inhibited inflammation by reshaping gut microbiota and metabolome

**Fig 6:**
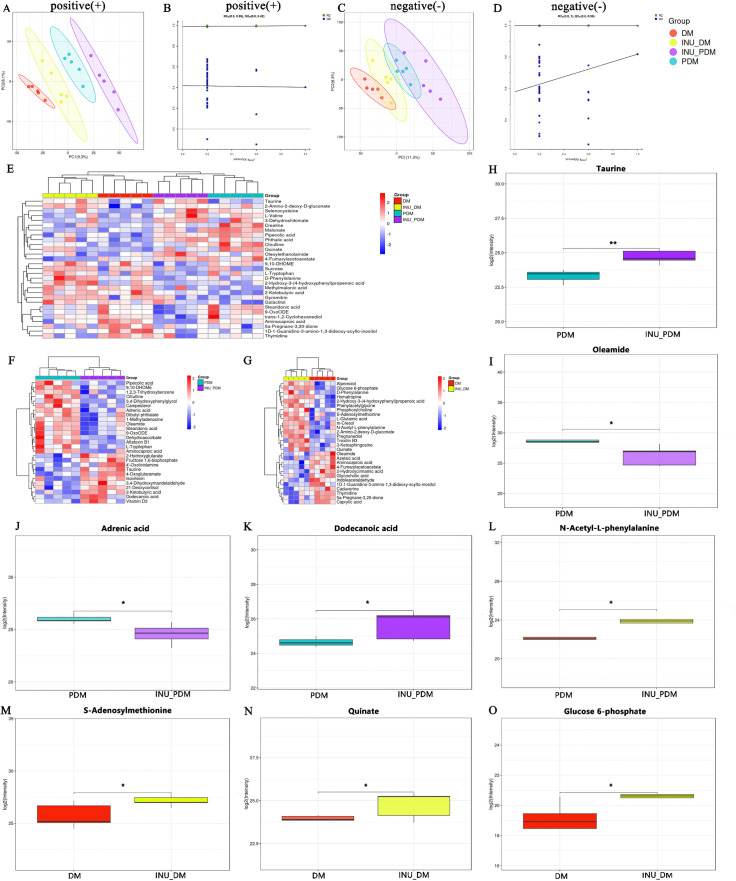
The effect of inulin treatment on plasma untargeted metabolomic profiles in mice of diverse groups. A and B: PLS-DA score plots and corresponding response permutation test plots in positive ion mode. C and D: PLS-DA score plots and corresponding response permutation test plots in negative ion mode. E: Heatmap of differential metabolites in four groups. F: Heatmap of PDM vs INU/PDM. G: Heatmap of DM vs INU/DM. Color key in the heat map represents the metabolite expression value: red represents the significant increases and blue stands for the remarkable decreases. H: Taurine. I: Oleamide. J: Adrenic acid. K: Dodecanoic acid. L: N-Acetyl-L-phenylalanine. M: S-Adenosylmethionine. N: Quinate. O: Glucose 6-phosphate. n = 5 per group;Wilcox rank-sum test.

Since inulin serve as a classic dietary fiber that can significantly affect the intestinal microecology, multi-omics analysis including untargeted metabolomics and 16S rRNA sequencing were further investigated. As a result, untargeted metabolomics analysis identified a total of 342 metabolites in the plasma samples. To objectively identify differential metabolites, we employed partial least squares discriminant analysis (PLS-DA) multivariate statistical methods to visualize and quantify groups separation (Fig 6A–6D). Fig 6A and 6C illustrated the difference among groups. Corresponding response permutation test plots (Fig 6B and 6D) indicated that the models were appropriate and reliable for prediction. The heatmap of metabolites revealed 29 differential metabolites in the four groups (Fig 6E). Considering the distinct glycemic and neuropathic characteristics associated with prediabetes and diabetes states, we further conducted a comparative analysis of metabolites profiles between intervention and non-intervention groups within each stage. Notably, inulin supplementation significantly altered stage-specific metabolic patterns, as demonstrated by the distinct heatmap profiles in both the prediabetic (Fig 6F) and diabetic (Fig 6G) stages. In a prediabetic stage, inulin treatment observably increased taurine (Fig 6H) and dodecanoic acid levels (Fig 6I), while downregulated oleamide and adrenic acid (Fig 6J and 6K). Similarly, our findings indicated a significant increase in the levels of N-Acetyl-L-phenylalanine, S-Adenosylmethionine, quinate and glucose 6-phosphate in the inulin intervention group compared to those observed during the diabetic stage (Fig 6L–6O). Consequently, our untargeted metabolomics data confirmed that inulin intervention possess the potential to modulate the metabolome compositions at the diverse stages of diabetic mice.

**Fig 7:**
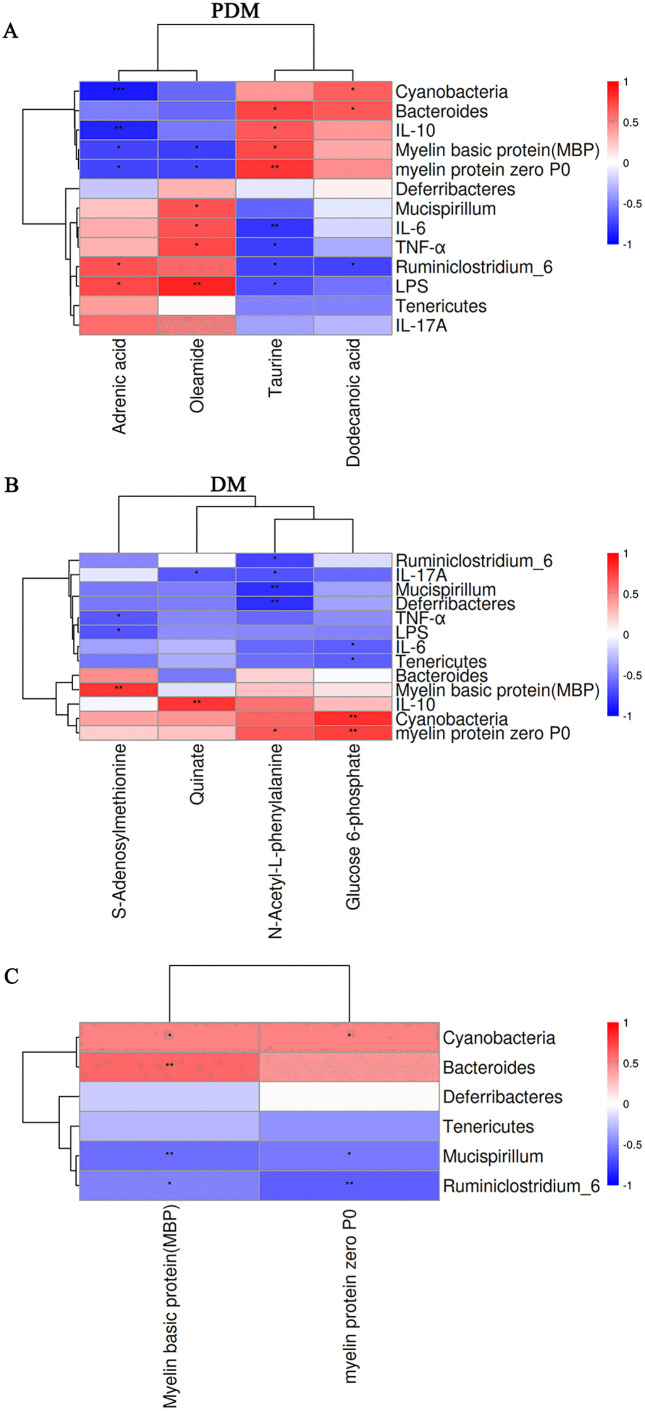
The associations among pro-inflammatory/anti-inflammatory cytokines, myelin proteins, differential gut microbiota and its metabolites. A and B showing the correlation analysis of significantly altered metabolites, differential fecal microbiota, inflammatory factors and myelin proteins after inulin intervention in the prediabetic and diabetic stages, respectively. C: The correlation analysis between myelin proteins levels and differential gut microbiota. *P < 0.05, **P < 0.01, ***P < 0.001.

To illustrate the composition of feccal microbiota, we performed 16S ribosomal RNA pyrosequencing. During the prediabetic and diabetic stages, inulin supplementation increased the relative abundance of *Cyanobacteria* with anti-diabetic effect, as well as decreased the relative abundances of pathogenic bacteria *Deferribacteres* and *Tenericutes* at phylum level. Similarly, at the genus level, inulin intervention upregulated the relative abundance of *Bacteroides* and downregulated the relative abundances of pathogenic *Ruminclostridium_6* and *Mucispirillum* (S2 Fig). In our present study, these changes in fecal microbiota were closely related to microbial metabolites, inflammatory factors, and indicators of peripheral neuropathy (Fig 7). In the prediabetic stage (Fig 7A), taurine was positively correlated with *Bacteroides*, MBP, P0 and IL-10, while negatively correlated with pro-inflammatory bacterium *Ruminiclostridium_6*, pro-inflammatory IL-6, TNF-α and LPS. We found that adrenic acid was positively correlated with LPS and pro-inflammatory bacteria *Ruminiclostridium_6*, while negatively correlated with *Cyanobacteria*, MBP, P0 and anti-inflammatory IL-10. Oleamide was positively correlated with pathogenic bacterium *Mucispirillum*, pro-inflammatory IL-6, TNF-α and LPS, but was negatively correlated with MBP and P0. Dodecanoic acid showed positively associated with *Bacteroides* and *Cyanobacteria,* and negatively associated with pro-inflammatory bacterium *Ruminiclostridium_6*. In parallel, inulin intervention significantly increased 4 metabolites in diabetes groups (Fig 7B). S-Adenosylmethionine, a methyl donor, was also positively associated with MBP and inversely associated with TNF-α and LPS. N-acetyl-L-phenylalanine and glucose 6-phosphate were positively related to P0 and/or anti-inflammatory bacterium *Cyanobacteria,* and inversely related to IL-17A, IL-6, and/or pro-inflammatory bacteria (*Ruminiclostridium_6,* *Deferribacteres* and *Tenericutes*), respectively. Additionally, quinate was negatively correlated with IL-17A and positively associated with IL-10. These results suggested that the interaction of 8 metabolic products (taurine, adrenic acid, oleamide, dodecanoic acid, S-Adenosylmethionine, N-acetyl-L-phenylalanine, quinate and glucose 6-phosphate) derived from gut microbiota (*Bacteroides*, *Cynobacteria*, *Mucispirillum,* *Ruminiclostridium_6,* *Deferribacteres* and *Tenericutes*) may probably be the vital elements of inulin effectiveness on DPN.

Remarkably, MBP showed a positive correlation with the relative abundances of beneficial bacteria, specifically *Cyanobacteria* and *Bacteroides.* Conversely, MBP displayed a negative correlation with the relative abundances of pathogenic bacteria consisting of *Mucispirillum* and *Ruminiclostridium_6* (Fig 7C). Similarly, P0 was positively associated with the relative abundance of *Cyanobacteria* and negatively associated with the relative abundance of pathogenic bacteria, including *Mucispirillum* and *Ruminiclostridium_6* (Fig 7C).

## 5. Discussion

In this study, leptin receptor mutant mice (*db/db* mice) served as a murine model of DPN to explore the underlying mechanisms responsible for therapeutic effect of inulin on DPN across diverse stages of diabetes. Our results demonstrated that the consumption of inulin effectively ameliorated peripheral nervous lesions at different stages, especially prediabetic stage. Notably, we further revealed that the underling mechanisms of inulin therapeutic efficacy was associated with repressing inflammatory response and restoring gut microecology by modulating gut microbiota and related metabolites. These results suggest that inulin may contribute to a new, cost-effective potential for prevention and treatment of DPN.

Inulin, a soluble dietary fiber, ferments in the colon and benefits for the growth and activity of beneficial microorganisms, promoting to digestive health and gut homeostasis [16], even administered at very low concentrations [34]. Both clinical [35] and rodent [36] studies have demonstrated that supplementation with an appropriate amount of inulin exerts an anti-diabetic effect through the modulation of gut microbiota. Our previous work also indicated that dietary inulin ameliorated glucose and lipid dysmetabolism in diverse stages of T2DM via suppressing inflammation and modulating gut microbiota, especially in prediabetic and early diabetic stages [24]. Consistently, in the present study, we found that the dietary fiber inulin may mitigate peripheral neuropathy at diverse stages of T2DM.

DPN is one of the most common complications in patients with T2DM. Most patients report sensory symptoms, including severe pain, burning sensations, numbness in the feet and legs, and even sensory loss. More than half of patients were highly dissatisfied with the conventional treatment aimed at reducing oxidative stress and improving blood circulation. The unclear understanding of the pathogenesis of DPN poses a remarkable barrier to the prevention and treatment efficiency. In the context of peripheral neuropathy, our study demonstrated that inulin intervention effectively attenuated mechanical allodynia and thermal hyperalgesia across different stages of diabetic mice, with particularly notable effects observed in the prediabetic stage. Furthermore, this therapeutic approach was also effective in significantly improving nerve conduction indicators. Meanwhile, IENFD acting as another critical biomarker for the diagnosis and investigation of neuropathy were quantitatively identified in small nerve fibers of the shin for the assessment of inulin effectiveness. Abnormal IENFD leads to sensory dysfunction and pain. Markedly boosted expression of PGP9.5^+^ IENFD in inulin-treated prediabetic stage indicated the critical role of inulin in peripheral neuroprotection.

To further explore the explanation for the protection of inulin against peripheral neuropathy damage, we systematically analyzed alterations in myelin protein expression within sciatic nerves of prediabetic and diabetic murine models. This investigation was motivated by the established pathological hallmark of diabetic peripheral neuropathy (DPN) progression – progressive thinning and segmental loss of periaxonal myelin sheaths, which directly impaired saltatory conduction and reduced nerve conduction velocity (NCV). Within the peripheral nervous system, nerve fibers are wrapped in a myelin sheath, which serves to protect and nourish the nerve fibers while facilitating the efficient transmission of nerve impulses [37]. P0, a Schwann cell-derived myelin marker, make up more than 50%−60% of the total protein in peripheral nervous systems myelin and plays a crucial role in the formation, stability, and maintenance of the myelin sheath. Additionally, MBP, another major component of the myelin sheath, is widely recognized as a myelin marker. Therefore, reduced expression of these proteins can negatively affect myelination [38,39]. In this study, the enhancement in MBP and P0 expressions in the sciatic nerve during the perdiabetic and diabetic stages with inulin supplementation indicated that inulin exhibited neuroprotective effects against diabetic peripheral nerve injury through preserving myelin sheaths integrity by myelin protein stabilization.

Dysbiosis of gut microorganisms leads to excessive LPS derived from Gram-negative pathogenic bacteria translocation from impeded gut barrier to blood circulation for triggering inflammation via stimulating TLR4 signalling, which ultimately may be implicated in the pathogenesis of DPN [40,41]. Increasing evidence has confirmed that a close association between the biomarkers of systemic inflammation and the progression of DPN progression [9]. Moreover, the anti-inflammation properties of inulin were mechanistically linked to modulation of LPS-TLR4-Macrophage signaling axis [42]. In parallel, an anti-inflammatory effect of inulin administration on DPN in diverse stages of diabetes was unveiled in this study by reducing pro-inflammatory LPS, IL-6, TNF-α and IL-17A and elevating anti-inflammatory IL-10. IL6 and TNF-α plays an important role in the development of inflammation-mediated diabetes and neuropathy [43]. IL-17A exacerbates neuropathy via activating immune cells, creating pro-inflammatory micro-environments and increasing oxidative stress [44]. IL-10 represents a pleiotropic cytokine with immunoregulatory functions, including suppression of pro-inflammatory cytokines production and modulation of innate/adaptive immune cell activity through the inhibition of antigen-presenting cells, differentiation of regulatory T cells and alternatively activated macrophages [45]. Moreover, observed negative correlations of MBP and P0 with pro-inflammatory IL-6, IL-17A, TNF-α and LPS suggested that the inhibition of inflammation may participate in myelination. Consequently, further research on the neuroprotective mechanisms of inulin should focus on examining inflammatory immune cells such as macrophages, regulatory T (Treg) cells.

Gut microbiota-peripheral nervous (PN) axis may act critical role in the occurrence and development of DPN [46]. In this study, inulin intervention significantly modulated the gut microbiota by increasing the relative abundance of *Bacteroides* and *Cyanobacteria*, while reducing *Deferribacteres*, *Tenericutes*, *Ruminclostridium_6* and *Mucispirillum.* These alterations suggest that inulin may attenuate DPN via modulation of gut-PN axis. It has been suggested that *Bacteroides* can help regulate blood glucose and lipid levels by influencing bile acid metabolism [47]. *Cyanobacteria*, a group of photosynthetic bacteria commonly found in aquatic and terrestrial environments, have gained attention in recent research for their potential effects on blood glucose and lipids. Report has shown that cyanobacterial bioactive compounds possess antimicrobial properties [48]. Furthermore, *Mucispirillum* is a pathogenic bacterium that inhabits the intestinal mucus layer. Myelination, an important component of PN, exhibits a close correation with gut microbiota. A recent study established that *db/db* mice colonized with gut microbiota from patients with diabetic sensorimotor peripheral neuropathy (DSPN) exhibited exacerbated peripheral neuropathy and significantly reduced MBP level compared to the control groups [9]. In germ-free mice, colonization with fast growth babies fecal bacteria markedly enhance MBP expression level [49]. Similarly, our results also exhibited that the effectiveness of inulin may attributed to the solid relationship between myelin proteins and differential bacteria by enhancing the expressions of both MBP and P0 that are two critical myelin proteins in myelination of PN.

Gut microbiota exerts its effects through its metabolites [50]. Our untargeted metabolomics analysis identified eight diabetes stage-specific metabolites associated with the intervention of inulin in diabetic peripheral neuropathy (DPN), suggesting that the protective effect of inulin against DPN across different diabetic stages is linked to metabolic reprogramming. We speculate that inulin mitigates systemic inflammation and facilitates neurological repair via regulating gut microbiota and their metabolic products. In the prediabetic stage, inulin supplementation significantly increased taurine, a metabolite known to enhance insulin sensitivity, exert anti-inflammatory effects, and confer neuroprotection [51], as well as dodecanoic acid (lauric acid), a medium-chain fatty acid with antidiabetic and antioxidant properties [52]. In contrast, inulin reduced levels of oleamide and adrenic acid. While oleamide is traditionally known for its sleep-inducing properties, it also exhibits pro-inflammatory effects by driving M0 macrophages toward an M1 phenotype and activating the NLRP3 inflammasome to induce IL-1β secretion [53]. Similarly, adrenic acid, an ω-6 polyunsaturated fatty acid, serves as a critical mediator of inflammation [54]. The inulin-induced reduction in both metabolites likely reflects a beneficial reprogramming of lipid metabolism, shifting the host toward an anti-inflammatory and neuroprotective state. During the diabetes, inulin intervention increased distinct metabolites including methylation (S-Adenosylmethionine, SAM) [55], glucose homeostasis (glucose 6-phosphate) [56], phenylalanine metabolic derivative N-acetyl-L-phenylalanine [57] and quinate (shown with anti-diabetic effort) [58]. Meanwhile, these metabolites changes were associated with favorable microbiota and suppression of pro-inflammatory indicators (e.g., LPS, TNF-α, IL-6), leading to the gut microbiota-metabolites-inflammation-PN axis is involved in the efficacy of inulin on diverse stage of diabetic with DPN. Furthermore, our lab is ongoing to functionally verify targeted interventions and underlying mechanisms involving differential bacteria and associated crucial metabolites in multi-antibiotic treated germ-free mice. In this study, we recognize that sexual dimorphism may influence the pathogenesis of diabetic neuropathy and the host response to dietary intervention. Consequently, we consider the inclusion of male mice serving as a valuable direction in future research. Investigating whether similar microbiota-metabolite-neuroinflammatory pathways are modulated in male models may reveal sex-specific mechanisms and broaden the translational relevance of inulin supplementation.

Considering the above, early dietary intervention with inulin supplementation at the prediabetic stage may mitigate the progression of neuropathy by targeting reversible metabolic and inflammatory pathways, prior to the onset of irreversible structural nerve damage. Inulin is recommended to be included in the diabetic diet as functional foods (e.g., fortified bread, yogurt) or supplements at doses of 10–20 g/day aligning with Food and Drug Administration/ European Food Safety Authority (FDA/EFSA) recommendations for daily fiber intake [16]. Its integration into clinical practice serving as preventive and therapeutic agent on the progression of clinical diabetic neuropathy via gut microbiota-metabolites-inflammation axis still needs further clinical verification.

## 6. Conclusion

We highlight that dietary inulin exhibits a peripheral neuroprotective efficacy in female *db/db* mice via reshaping gut homeostasis and suppressing inflammation, especially during the prediabetic stage. This findings might help to provide new preventive and therapeutic strategies for DPN in diverse stages of female diabetic patients.

## Supporting information

S1 Table The changes in basic metabolic indicators of mice at diverse stages of diabetes with peripheral neuropathy during the inulin treatment. Upon completion of the administration period, inulin was found to mitigate body weight gain, hyperglycemcia, and dyslipidmia, in addition to enhancing insulin levels in murine models of prediabetes and diabetes. However, the intervention did not demonstrate a statistically different effect on ad libitum water intake and cumulative food consumption during the same timeframe. n = 5–10 per group. Differences between inulin-treated and untreated groups during the same stage were analyzed using an unpaired Student’s t-test or Mann-Whitney U test. *P < 0.05 and **P < 0.01 compared with the PDM group; #*P* < 0.05 and ##*P* < 0.01 compared with the DM group; and ns: no significance.
(DOC)

S1 Fig Diabetic peripheral neuropathy model was established. A: The von Frey test was used to assess the onset of mechanical allodynia curve for mice over time. B: The paw withdraw latency was recorded as thermal hyperalgesia over time. The paw withdrawal mechanical threshold (A) and paw withdrawal thermal latency (B) were measured at different time points (week 1, 2, 3, 4, 5, 6). C: Representative traces of compound action potential (CAP) of sciatic nerve in each model from different stages. D: Conduct velocity (CV) expression of sciatic nerve in each model at various disease stages. E: In comparison to control group, sensory nerve action potential (SNAP) expression in model group with diverse stages of disease. Data are expressed as mean ± SEM; n = 5 per group. Statistical significance in Figure (A-B) and Figure (D-E) was determined with tow-way ANOVA and one-way ANOVA, respectively. *P < 0.05 and ***P < 0.001 PDM group compared with the control group; ##*P* < 0.01 and ###*P* < 0.001 DM group compared with the control group.
(TIF)

S2 Fig Heatmap analysis of microbial species at the phylum level and genus level in diverse stages of T2DM mice feces. A: The relative abundance of microbial species at the phylum level in the feces of mice. B: The relative abundance of microbial species at the genus level in the feces of mice. n = 5/group.
(TIF)

S1 File All data. (ZIP)

## Abbreviations

DPN: Diabetic peripheral neuropathy
T2DM: Type 2 diabetes mellitus
LPS: Lipopolysaccharide
TLR-4: Toll-like receptor-4
IL: Interleukin
TNF-α: Tumor necrosis factor-α
INU: Inulin
PDM: Prediabetes
DM: Diabetes mellitus
PWT: Paw withdrawal threshold
PWL: Paw withdraw latency
SNCV: Sensory nerve conduction velocities
SNAP: Sensory nerve action potential
MBP: Myelin basic protein
P0: Myelin protein zero
IENFD: Intraepidermal nerve fiber density
PGP 9.5: Protein gene product 9.5
CBA: Cytometric bead array
LC/MS: Liquid chromatography/mass spectrometry
RDA: Redundancy analysis
PLS-DA: Partial least squares-discriminant analysis
STZ: Streptozotocin
DSPN: Diabetic sensorimotor peripheral neuropathy
SAM: S-Adenosylmethionine
FDA/EFSA: Food and Drug Administration/ European Food Safety Authority
