# Supplementary material for: Dietary fiber inulin mitigates peripheral neuropathy in different stages of diabetes via modulating gut microbiota and metabolites in db/db mice
Source: PLoS One. 2025 Nov 20;20(11):e0336962. doi: 10.1371/journal.pone.0336962 (PMC12633925; doi:10.1371/journal.pone.0336962)
Supplement: S1 Table — Upon completion of the administration period, inulin was found to mitigate body weight gain, hyperglycemcia, and dyslipidmia, in addition to enhancing insulin levels in murine models of prediabetes and diabetes. However, the intervention did not demonstrate a statistically different effect on ad libitum water intake and cumulative food consumption during the same timeframe. n = 5–10 per group. Differences between inulin-treated and untreated groups during the same stage were analyzed using an unpaired Student’s t-test or Mann-Whitney U test. *P < 0.05 and **P < 0.01 compared with the PDM group; #P < 0.05 and ##P < 0.01 compared with the DM group; and ns: no significance. (DOC) [file pone.0336962.s001.doc]

**Supplementary 1 (S1) Table**

Effect of inulin on the fundamental metabolic indicators in diverse stages of diabetic *db/db* mice.

| biochemical markers | PDM | INU/PDM | DM | INU/DM |
| --- | --- | --- | --- | --- |
| Food intake (g/d) | 5.400±0.436 | 5.267±0.252^ns^ | 6.133±0.058 | 5.633±0.351^ns^ |
| Water intake(mL/d) | 11.200±1.179 | 8.633±2.113^ns^ | 12.400±3.291 | 9.357±2.846^ns^ |
| Body weight(g) | 49.350±1.797 | 46.640±1.503** | 44.850±3.191 | 48.800±3.414^#^ |
| Blood glucose(mmol/L) | 23.030±3.246 | 17.580±3.885** | 27.140±1.547 | 24.620±1.782^##^ |
| Insulin(ng/mL) | 0.209±0.024 | 0.265±0.027** | 0.213±0.024 | 0.258±0.034^#^ |
| Glycated hemoglobin(ug/mL) | 54.450±2.191 | 48.210±2.003** | 86.460±1.976 | 83.480±1.623^#^ |
| Triglycerides(mmol/L) | 2.150±0.350 | 1.617±0.256* | 2.850±0.308 | 2.417±0.299^#^ |
| Total cholesterol(mmol/L) | 2.783±0.691 | 1.917±0.232* | 3.233±0.356 | 2.583±0.584^#^ |

Data are expressed as mean ±SEM, n=5-10/group. **P* < 0.05 and ***P* < 0.01 compared with the PDM group; #*P* < 0.05 and ##*P* < 0.01 compared with the DM group; and ns: no significance.
