# Supplementary material for: Dietary fiber inulin mitigates peripheral neuropathy in different stages of diabetes via modulating gut microbiota and metabolites in db/db mice
Source: PLoS One. 2025 Nov 20;20(11):e0336962. doi: 10.1371/journal.pone.0336962 (PMC12633925; doi:10.1371/journal.pone.0336962)
Supplement: S1 File — (ZIP) [file pone.0336962.s004.zip › Datasets for Fig 3-5.doc]

Table 1. The data of Myelin basic protein levels (MBP).

| Group  Sample | PDM group | INU/PDM group | DM group | INU/DM group |
| --- | --- | --- | --- | --- |
| 1 | 2.1062 | 2.3549 | 1.9611 | 2.4279 |
| 2 | 2.1918 | 2.2632 | 1.9059 | 2.2506 |
| 3 | 2.0565 | 2.3435 | 2.1842 | 2.1429 |
| 4 | 2.2629 | 2.2473 | 2.1092 | 2.1555 |
| 5 | 2.2276 | 2.2627 | 1.9165 | 2.0999 |

Table 2. The data of myelin protein zero levels (P0).

| Group  Sample | PDM group | INU/PDM group | DM group | INU/DM group |
| --- | --- | --- | --- | --- |
| 1 | 2.3516 | 2.5622 | 2.2128 | 2.3302 |
| 2 | 2.4850 | 2.6270 | 2.2514 | 2.2290 |
| 3 | 2.3737 | 2.6654 | 2.1188 | 2.5917 |
| 4 | 2.3952 | 2.5379 | 2.0932 | 2.2493 |
| 5 | 2.5075 | 2.5337 | 1.9021 | 2.5214 |

Table 3. The data of inflammatory indicators.

| Group  cytokines | PDM group | INU/PDM group | DM group | INU/DM group |
| --- | --- | --- | --- | --- |
| IL-6 | 14.43 | 10.67 | 29.30 | 15.02 |
|  | 21.50 | 15.02 | 21.14 | 13.10 |
|  | 23.32 | 13.38 | 23.50 | 23.10 |
|  | 14.47 | 4.47 | 36.78 | 25.84 |
|  | 13.69 | 6.17 | 32.23 | 10.35 |
|  |  |  |  |  |
| TNF-α | 22.01 | 13.59 | 27.89 | 22.03 |
|  | 28.37 | 14.31 | 31.48 | 19.03 |
|  | 30.61 | 16.31 | 30.16 | 19.31 |
|  | 18.09 | 9.38 | 35.92 | 12.52 |
|  | 12.23 | 4.03 | 29.2 | 23.51 |
|  |  |  |  |  |
| IL-17A | 3.70 | 1.12 | 3.76 | 2.42 |
|  | 2.23 | 0.96 | 5.67 | 2.73 |
|  | 2.09 | 1.45 | 5.95 | 3.84 |
|  | 1.37 | 2.34 | 4.50 | 3.66 |
|  | 1.49 | 1.79 | 3.76 | 2.42 |
|  |  |  |  |  |
| IL-10 | 19.63 | 31.16 | 34.63 | 31.16 |
|  | 22.02 | 22.49 | 26.02 | 36.49 |
|  | 27.45 | 33.63 | 23.45 | 39.01 |
|  | 22.52 | 30.43 | 29.52 | 26.37 |
|  | 18.19 | 29.18 | 31.19 | 42.34 |
|  |  |  |  |  |
| LPS | 0.199695 | 0.160635 | 0.427925 | 0.39074 |
|  | 0.211055 | 0.157705 | 0.424725 | 0.37608 |
|  | 0.184825 | 0.170005 | 0.408390 | 0.409065 |
|  | 0.18765 | 0.182409 | 0.410392 | 0.348692 |
|  | 0.17927 | 0.160031 | 0.398410 | 0.380014 |

Table 4. The data of Intraepidermal nerve fiber density.

| Group  Sample | PDM group | INU/PDM group | DM group | INU/DM group |
| --- | --- | --- | --- | --- |
| 1 | 1.33 | 2.09 | 0.43 | 1.24 |
| 2 | 1.52 | 1.77 | 1.09 | 0.63 |
| 3 | 0.88 | 2.6 | 1.22 | 1.7 |
