# Supplementary material for: Dietary fiber inulin mitigates peripheral neuropathy in different stages of diabetes via modulating gut microbiota and metabolites in db/db mice
Source: PLoS One. 2025 Nov 20;20(11):e0336962. doi: 10.1371/journal.pone.0336962 (PMC12633925; doi:10.1371/journal.pone.0336962)
Supplement: S1 File — (ZIP) [file pone.0336962.s004.zip › Datasets for Supplementary 2 (S2) Fig.doc]

Table 1. The data of paw withdrawal threshold.

| Time(w)  CON group | 1w | 2w | 3w | 4w | 5w | 6w |
| --- | --- | --- | --- | --- | --- | --- |
| 1 | 1.40 | 1.40 | 2.00 | 2.00 | 1.40 | 1.40 |
| 2 | 2.00 | 2.00 | 1.40 | 2.00 | 1.40 | 1.40 |
| 3 | 1.40 | 2.00 | 1.40 | 1.40 | 1.40 | 1.40 |
| 4 | 2.00 | 1.40 | 2.00 | 1.40 | 2.00 | 2.00 |
| 5 | 1.40 | 1.40 | 2.00 | 1.40 | 2.00 | 1.40 |
| 6 | 2.00 | 2.00 | 2.00 | 1.40 | 1.40 | 2.00 |
| 7 | 2.00 | 2.00 | 1.40 | 2.00 | 1.40 | 1.40 |
| 8 | 2.00 | 1.40 | 1.40 | 2.00 | 2.00 | 2.00 |
| Time(w)  PDM group | 1w | 2w | 3w | 4w | 5w | 6w |
| 1 | 1.40 | 2.00 | 1.00 | 0.60 | 1.00 | 0.07 |
| 2 | 2.00 | 1.40 | 2.00 | 1.00 | 1.40 | 0.40 |
| 3 | 2.00 | 2.00 | 1.40 | 1.40 | 1.00 | 0.60 |
| 4 | 2.00 | 2.00 | 1.00 | 2.00 | 1.40 | 0.60 |
| 5 | 1.40 | 1.00 | 2.00 | 1.40 | 0.60 | 0.60 |
| 6 | 1.40 | 1.40 | 1.40 | 1.00 | 1.40 | 0.16 |
| 7 | 1.40 | 1.00 | 1.40 | 0.60 | 0.40 | 0.60 |
| 8 | 2.00 | 1.40 | 1.00 | 1.40 | 0.40 | 0.16 |
| Time(w)  DM group | 1w | 2w | 3w | 4w | 5w | 6w |
| 1 | 1.40 | 1.40 | 1.40 | 1.00 | 1.40 | 0.40 |
| 2 | 1.40 | 1.00 | 1.40 | 1.40 | 1.00 | 0.07 |
| 3 | 1.00 | 1.40 | 1.00 | 0.40 | 0.07 | 0.07 |
| 4 | 0.60 | 0.60 | 0.40 | 1.00 | 0.40 | 0.16 |
| 5 | 1.40 | 1.40 | 1.00 | 0.40 | 0.07 | 0.16 |
| 6 | 1.00 | 1.40 | 1.00 | 0.16 | 0.16 | 0.07 |
| 7 | 1.40 | 1.00 | 1.00 | 0.40 | 0.16 | 0.07 |
| 8 | 1.40 | 0.60 | 1.00 | 0.40 | 0.16 | 0.07 |

Table 2. The data of paw withdrawal thermal latency.

| Time(w)  CON group | 1w | 2w | 3w | 4w | 5w | 6w |
| --- | --- | --- | --- | --- | --- | --- |
| 1 | 12.0 | 12.0 | 12.0 | 12.0 | 12.0 | 12.0 |
| 2 | 12.0 | 12.0 | 11.0 | 12.0 | 12.0 | 11.0 |
| 3 | 12.0 | 12.0 | 12.0 | 12.0 | 12.0 | 12.0 |
| 4 | 11.0 | 12.0 | 12.0 | 12.0 | 12.0 | 12.0 |
| 5 | 12.0 | 12.0 | 12.0 | 12.0 | 12.0 | 12.0 |
| 6 | 12.0 | 11.0 | 11.0 | 11.0 | 12.0 | 12.0 |
| 7 | 12.0 | 12.0 | 12.0 | 12.0 | 11.0 | 12.0 |
| 8 | 12.0 | 12.0 | 11.0 | 11.0 | 12.0 | 12.0 |
| Time(w)  PDM group | 1w | 2w | 3w | 4w | 5w | 6w |
| 1 | 11.0 | 12.0 | 10.0 | 10.0 | 6.0 | 9.0 |
| 2 | 10.3 | 11.0 | 11.0 | 9.0 | 8.0 | 6.0 |
| 3 | 10.9 | 10.2 | 9.0 | 8.0 | 6.7 | 5.3 |
| 4 | 11.0 | 11.3 | 10.0 | 10.0 | 8.7 | 7.0 |
| 5 | 11.8 | 10.0 | 10.0 | 11.0 | 7.4 | 10.0 |
| 6 | 11.0 | 11.0 | 11.0 | 9.7 | 10.3 | 9.0 |
| 7 | 11.0 | 11.0 | 10.3 | 10.5 | 9.0 | 6.7 |
| 8 | 12.0 | 9.1 | 11.0 | 9.4 | 6.3 | 3.4 |
| Time(w)  DM group | 1w | 2w | 3w | 4w | 5w | 6w |
| 1 | 10.5 | 10.0 | 10.0 | 8.3 | 5.6 | 2.7 |
| 2 | 10.3 | 10.0 | 11.0 | 7.0 | 3.4 | 2.2 |
| 3 | 11.0 | 10.0 | 8.0 | 8.0 | 4.8 | 3.5 |
| 4 | 11.7 | 11.0 | 9.0 | 6.3 | 7.8 | 4.7 |
| 5 | 11.0 | 11.0 | 11.0 | 4.3 | 4.9 | 3.8 |
| 6 | 11.0 | 11.0 | 10.0 | 5.8 | 5.4 | 2.3 |
| 7 | 11.0 | 12.0 | 10.0 | 3.4 | 3.2 | 2.7 |
| 8 | 12.0 | 11.0 | 9.0 | 6.2 | 3.7 | 4.5 |

Table 3. The data of sensory nerve conduct velocity.

| Group  Sample | CON group | DM group | INU/DM group |
| --- | --- | --- | --- |
| 1 | 25.54 | 24.05 | 22.66 |
| 2 | 29.62 | 26.77 | 24.89 |
| 3 | 29.23 | 25.03 | 22.93 |
| 4 | 32.92 | 28.00 | 23.95 |
| 5 | 30.77 | 26.15 | 25.27 |

Table 4. The data of sensory nerve action potential.

| Group  Sample | CON group | DM group | INU/DM group |
| --- | --- | --- | --- |
| 1 | 42.54 | 35.78 | 29.66 |
| 2 | 39.62 | 33.49 | 34.89 |
| 3 | 37.23 | 35.69 | 32.93 |
| 4 | 33.92 | 36.62 | 26.95 |
| 5 | 39.77 | 32.58 | 31.27 |
