# Supplementary material for: Dietary fiber inulin mitigates peripheral neuropathy in different stages of diabetes via modulating gut microbiota and metabolites in db/db mice
Source: PLoS One. 2025 Nov 20;20(11):e0336962. doi: 10.1371/journal.pone.0336962 (PMC12633925; doi:10.1371/journal.pone.0336962)
Supplement: S1 File — (ZIP) [file pone.0336962.s004.zip › Datasets for Fig 1.doc]

Table 1. The data of paw withdrawal threshold.

| Time(w)  PDM group | 1w | 2w | 3w | 4w | 5w | 6w |
| --- | --- | --- | --- | --- | --- | --- |
| 1 | 1.40 | 2.00 | 1.00 | 0.60 | 1.00 | 0.07 |
| 2 | 2.00 | 1.40 | 2.00 | 1.00 | 1.40 | 0.40 |
| 3 | 2.00 | 2.00 | 1.40 | 1.40 | 1.00 | 0.60 |
| 4 | 2.00 | 2.00 | 1.00 | 2.00 | 1.40 | 0.60 |
| 5 | 1.40 | 1.00 | 2.00 | 1.40 | 0.60 | 0.60 |
| 6 | 1.40 | 1.40 | 1.40 | 1.00 | 1.40 | 0.16 |
| 7 | 1.40 | 1.00 | 1.40 | 0.60 | 0.40 | 0.60 |
| 8 | 2.00 | 1.40 | 1.00 | 1.40 | 0.40 | 0.16 |
| 9 | 1.40 | 2.00 | 1.40 | 1.00 | 1.40 | 0.60 |
| 10 | 1.40 | 2.00 | 1.40 | 1.40 | 1.00 | 0.16 |
| Time(w)  INU/PDM  group | 1w | 2w | 3w | 4w | 5w | 6w |
| 1 | 2.00 | 0.60 | 1.40 | 1.00 | 0.60 | 0.60 |
| 2 | 1.40 | 2.00 | 1.40 | 1.40 | 1.00 | 0.60 |
| 3 | 1.40 | 2.00 | 2.00 | 1.40 | 2.00 | 1.40 |
| 4 | 2.00 | 2.00 | 2.00 | 2.00 | 1.00 | 1.00 |
| 5 | 2.00 | 1.40 | 1.40 | 1.40 | 1.40 | 0.40 |
| 6 | 2.00 | 1.40 | 2.00 | 1.40 | 1.40 | 0.60 |
| 7 | 1.40 | 2.00 | 1.40 | 1.40 | 1.40 | 1.40 |
| 8 | 2.00 | 1.40 | 1.40 | 1.40 | 1.00 | 1.40 |
| 9 | 1.40 | 2.00 | 1.40 | 2.00 | 2.00 | 2.00 |
| 10 | 2.00 | 2.00 | 1.00 | 1.40 | 1.40 | 1.00 |
| Time(w)  DM group | 1w | 2w | 3w | 4w | 5w | 6w |
| 1 | 1.40 | 1.40 | 1.40 | 1.00 | 1.40 | 0.40 |
| 2 | 1.40 | 1.00 | 1.40 | 1.40 | 1.00 | 0.07 |
| 3 | 1.00 | 1.40 | 1.00 | 0.40 | 0.07 | 0.07 |
| 4 | 0.60 | 0.60 | 0.40 | 1.00 | 0.40 | 0.16 |
| 5 | 1.40 | 1.40 | 1.00 | 0.40 | 0.07 | 0.16 |
| 6 | 1.00 | 1.40 | 1.00 | 0.16 | 0.16 | 0.07 |
| 7 | 1.40 | 1.00 | 1.00 | 0.40 | 0.16 | 0.07 |
| 8 | 1.40 | 0.60 | 1.00 | 0.40 | 0.16 | 0.07 |
| 9 | 1.00 | 1.00 | 0.40 | 0.60 | 0.16 | 0.02 |
| 10 | 1.00 | 1.40 | 1.00 | 0.40 | 0.07 | 0.07 |
| Time(w)  INU/DM group | 1w | 2w | 3w | 4w | 5w | 6w |
| 1 | 1.40 | 1.40 | 1.40 | 1.00 | 1.00 | 0.60 |
| 2 | 1.40 | 1.40 | 1.40 | 1.00 | 0.60 | 0.40 |
| 3 | 1.40 | 1.40 | 1.00 | 1.00 | 1.00 | 0.40 |
| 4 | 1.00 | 1.00 | 1.40 | 1.00 | 1.00 | 1.40 |
| 5 | 1.40 | 1.00 | 1.00 | 0.60 | 0.60 | 0.40 |
| 6 | 1.40 | 1.40 | 1.00 | 0.60 | 0.60 | 1.00 |
| 7 | 1.00 | 1.00 | 1.00 | 0.60 | 0.40 | 1.00 |
| 8 | 1.40 | 1.40 | 1.00 | 1.00 | 0.60 | 0.40 |
| 9 | 0.60 | 1.00 | 1.00 | 0.40 | 1.00 | 0.40 |
| 10 | 1.40 | 1.40 | 1.40 | 2.00 | 0.60 | 0.40 |

Table 2. The data of paw withdrawal thermal latency.

| Time(w)  PDM group | 1w | 2w | 3w | 4w | 5w | 6w |
| --- | --- | --- | --- | --- | --- | --- |
| 1 | 11.0 | 12.0 | 10.0 | 10.0 | 6.0 | 9.0 |
| 2 | 10.3 | 11.0 | 11.0 | 9.0 | 8.0 | 6.0 |
| 3 | 10.9 | 10.2 | 9.0 | 8.0 | 6.7 | 5.3 |
| 4 | 11.0 | 11.3 | 10.0 | 10.0 | 8.7 | 7.0 |
| 5 | 11.8 | 10.0 | 10.0 | 11.0 | 7.4 | 10.0 |
| 6 | 11.0 | 11.0 | 11.0 | 9.7 | 10.3 | 9.0 |
| 7 | 11.0 | 11.0 | 10.3 | 10.5 | 9.0 | 6.7 |
| 8 | 12.0 | 9.1 | 11.0 | 9.4 | 6.3 | 3.4 |
| 9 | 12.0 | 11.0 | 12.0 | 8.7 | 8.8 | 8.1 |
| 10 | 12.0 | 11.0 | 10.0 | 10.9 | 8.5 | 4.9 |
| Time(w)  INU/PDM group | 1w | 2w | 3w | 4w | 5w | 6w |
| 1 | 12.0 | 10.0 | 11.6 | 10.9 | 8.0 | 10.0 |
| 2 | 11.3 | 11.0 | 11.0 | 9.0 | 9.3 | 8.0 |
| 3 | 11.4 | 12.0 | 11.0 | 10.0 | 11.3 | 11.0 |
| 4 | 11.7 | 11.0 | 9.0 | 10.3 | 11.0 | 7.8 |
| 5 | 11.0 | 12.0 | 11.0 | 11.0 | 10.0 | 10.8 |
| 6 | 12.0 | 12.0 | 11.5 | 10.0 | 11.0 | 10.0 |
| 7 | 10.0 | 11.3 | 11.0 | 11.0 | 11.0 | 8.2 |
| 8 | 12.0 | 11.0 | 11.4 | 12.0 | 10.7 | 7.9 |
| 9 | 11.0 | 12.0 | 12.0 | 10.5 | 7.2 | 8.0 |
| 10 | 12.0 | 11.0 | 12.0 | 10.5 | 8.7 | 10.2 |
| Time(w)  DM group | 1w | 2w | 3w | 4w | 5w | 6w |
| 1 | 10.5 | 10.0 | 10.0 | 8.3 | 5.6 | 2.7 |
| 2 | 10.3 | 10.0 | 11.0 | 7.0 | 3.4 | 2.2 |
| 3 | 11.0 | 10.0 | 8.0 | 8.0 | 4.8 | 3.5 |
| 4 | 11.7 | 11.0 | 9.0 | 6.3 | 7.8 | 4.7 |
| 5 | 11.0 | 11.0 | 11.0 | 4.3 | 4.9 | 3.8 |
| 6 | 11.0 | 11.0 | 10.0 | 5.8 | 5.4 | 2.3 |
| 7 | 11.0 | 12.0 | 10.0 | 3.4 | 3.2 | 2.7 |
| 8 | 12.0 | 11.0 | 9.0 | 6.2 | 3.7 | 4.5 |
| 9 | 10.2 | 10.4 | 9.0 | 7.9 | 4.0 | 4.9 |
| 10 | 9.7 | 10.2 | 7.0 | 8.3 | 4.2 | 5.0 |
| Time(w)  INU/DM group | 1w | 2w | 3w | 4w | 5w | 6w |
| 1 | 11.0 | 11.0 | 10.0 | 7.8 | 7.3 | 6.8 |
| 2 | 11.0 | 12.0 | 11.0 | 8.6 | 6.9 | 6.3 |
| 3 | 11.0 | 11.0 | 10.0 | 7.8 | 6.7 | 6.4 |
| 4 | 11.0 | 11.0 | 11.0 | 7.3 | 7.7 | 7.4 |
| 5 | 12.0 | 12.0 | 11.0 | 9.7 | 8.5 | 7.8 |
| 6 | 12.0 | 11.0 | 11.0 | 10.0 | 8.8 | 6.1 |
| 7 | 12.0 | 12.0 | 11.0 | 7.9 | 7.3 | 6.4 |
| 8 | 12.0 | 12.0 | 11.0 | 8.9 | 7.6 | 7.3 |
| 9 | 12.0 | 11.0 | 10.2 | 8.7 | 10.3 | 3.0 |
| 10 | 11.2 | 12.0 | 11.0 | 9.0 | 6.7 | 3.5 |

Table 3. The data of sensory nerve conduct velocity.

| Group  Sample | PDM group | INU/PDM group | DM group | INU/DM group |
| --- | --- | --- | --- | --- |
| 1 | 24.05 | 27.54 | 22.66 | 27.98 |
| 2 | 26.77 | 29.62 | 24.89 | 27.58 |
| 3 | 25.03 | 27.23 | 22.93 | 26.94 |
| 4 | 28.00 | 30.13 | 23.95 | 25.47 |
| 5 | 26.15 | 29.05 | 25.27 | 29.32 |

Table 4. The data of sensory nerve action potential.

| Group  Sample | PDM group | INU/PDM group | DM group | INU/DM group |
| --- | --- | --- | --- | --- |
| 1 | 35.78 | 38.14 | 29.66 | 37.42 |
| 2 | 33.49 | 41.67 | 34.89 | 35.17 |
| 3 | 35.69 | 37.57 | 32.93 | 37.43 |
| 4 | 36.62 | 39.01 | 26.95 | 38.94 |
| 5 | 32.58 | 35.23 | 31.27 | 39.32 |
